# Sleep in the Supine Position During Pregnancy is Associated with Fetal Cerebral Redistribution

**DOI:** 10.3390/jcm9061773

**Published:** 2020-06-07

**Authors:** Nicole Robertson, Satomi Okano, Sailesh Kumar

**Affiliations:** 1 Mater Research Institute—University of Queensland, Level 3 Aubigny Place, Raymond Terrace, South Brisbane QLD 4101, Queensland, Australia; n.t.brown@uqconnect.edu.au (N.R.); satomi.okano@qimrberghofer.edu.au (S.O.); 2 Mater Mothers’ Hospital, Raymond Terrace, South Brisbane QLD 4101, Queensland, Australia; 3 Faculty of Medicine, The University of Queensland, St Lucia, Brisbane QLD 4072, Australia; 4 Statistics Unit, QIMR Berghofer Medical Research Institute, Herston, Brisbane QLD 4006, Australia

**Keywords:** supine, sleep position, cerebral redistribution, cerebroplacental ratio, pregnancy

## Abstract

The supine sleep position in late pregnancy is a major risk factor for stillbirth, with a population attributable risk of 5.8% and one in four pregnant women reportedly sleeping in a supine position. Although the mechanisms linking the supine sleep position and late stillbirth remain unclear, there is evidence that it exacerbates pre-existing maternal sleep disordered breathing, which is another known risk factor for adverse perinatal outcomes. Given that maternal sleep position is a potentially modifiable risk factor, the aim of this study was to characterize and correlate uteroplacental and fetal hemodynamics, including cardiac function, in a cohort of women with apparently uncomplicated pregnancies with their nocturnal sleep position. This was a prospective observational cohort study at an Australian tertiary obstetric hospital. Women were asked to complete a series of questions related to their sleep position in late pregnancy after 35 weeks of completed gestation. They also underwent an ultrasound assessment where Doppler indices of various fetoplacental vessels and fetal cardiac function were measured. Regional cerebral perfusion was also assessed. Pregnancy outcome data was extracted from the electronic hospital database for analysis. A total of 274 women were included in the final analysis. Of these, 78.1% (214/274) reported no supine sleep, and 21.9% (60/274) reported going to sleep in a supine position. The middle cerebral artery, anterior cerebral artery, and vertebral artery pulsatility indices were all significantly lower in the supine sleep cohort, as was the cerebroplacental ratio. There were no significant differences in the mode or indication for delivery or in serious neonatal outcomes, including 5-min Apgar score < 7, acidosis, and neonatal intensive care unit admission between cohorts. Women in the supine cohort were more likely to have an infant with a BW > 90th centile (*p* = 0.04). This data demonstrates fetal brain sparing in association with the maternal supine sleep position in a low-risk population. This data contributes to the growing body of literature attempting to elucidate the etiological pathways responsible for the association of late stillbirth with the maternal supine sleep position.

## 1. Introduction

Epidemiological data suggests that up to one in three women in early pregnancy and one in five women in late pregnancy sleep in the supine position [1], and overall, almost 27% of pregnant women spend at least some time sleeping on their backs during the night [2]. Sleeping in the supine position is potentially causally implicated in late stillbirth [3,4,5,6,7,8], with almost 56% of women experiencing this complication believing that the time of fetal demise was at night [9]. Currently, it is estimated that the supine sleep position is associated with a 5.8% population attributable risk for late stillbirth [8], although a recent meta-analysis suggested the risk may be substantially higher at 37% [10]. This highlights the importance of maternal sleep position as a possible predisposing etiological cause, and importantly, as a potentially modifiable risk factor to reduce the risk of late stillbirth. Although the mechanisms linking the supine sleep position and late stillbirth remain unclear, there is evidence that sleeping in the supine position exacerbates pre-existing maternal sleep disordered breathing (SDB) [8,11], which is a known risk factor for adverse perinatal outcomes [12,13].

In the supine position, the inferior vena cava and aorta are compressed against the maternal spine by the heavy gravid uterus, which causes not only significant reduction in venous return to the maternal heart, but also diminution of aortic blood flow by almost 30% [14,15]. The subsequent reduction in maternal cardiac output results in a drop in uteroplacental perfusion, causing impaired fetal oxygenation and changes in fetal Doppler indices [16,17,18,19,20].

Given this background, the aim of this study was to prospectively characterize and correlate uteroplacental and fetal hemodynamics, including cardiac function, in a cohort of women with apparently uncomplicated pregnancies with their nocturnal sleep position.

## 2. Materials and Methods

This was a prospective observational study of women aged between 18 and 50 years, with non-anomalous fetuses and uncomplicated singleton pregnancies. To be eligible for inclusion, women were required to have a full medical history available and to have had their pregnancy dated within the first trimester. The study was undertaken at the Mater Mothers’ Hospital in Brisbane, Australia between July 2017 and June 2019. Relevant ethics, governance, and privacy approvals were obtained from the Mater Human Research Ethics Committee and Governance office, respectively (HREC/17/MHS/34). After informed written consent was obtained, all women underwent a single ultrasound assessment in late pregnancy (from 35 weeks of gestation) and completed a sleep questionnaire (Supplementary Table S1), which included specific questions relating to sleep position. The questionnaire asked women to describe their sleep position both going to sleep and waking from sleep before they were pregnant, over the last one month of pregnancy, over the last week of pregnancy, and the night prior to completing the questionnaire. Women were able to simply answer “yes” or “no” to each question and were also asked to quantify the amount of time they spent in each position as rarely (<10%), sometimes (10–50%), or mostly (>50%).

For the purposes of analysis, if women answered “yes,” “sometimes,” or “mostly” to question of going to sleep in the supine position at any time over the preceding month, week, or night of pregnancy, they were categorized into the supine cohort, whereas women who said “no” or “rarely” were classified as controls.

All ultrasound examinations were carried out using an Affinity 70G (Philips, USA) or a Voluson E8 (GE, Zipf, Austria) ultrasound machine by a single experienced sonographer (NR). The estimated fetal weight (EFW) was calculated using Hadlock’s formula [21]. Doppler parameters of various fetal vessels were measured, namely the middle cerebral artery pulsatility index (MCA PI), anterior cerebral artery pulsatility index (ACA PI), posterior cerebral artery pulsatility index (PCA PI), vertebral artery pulsatility index (VertA PI), umbilical artery pulsatility index (UA PI), and umbilical vein time averaged flow velocity (UV TAV). In addition, fetal cardiac output (CO) and maternal uterine artery pulsatility index (UtA PI) were measured. All measurements were recorded in triplicate over multiple cardiac cycles in the absence of maternal or fetal breathing movements with the average values reported.

The MCA, ACA, and PCA were identified in a transverse section of the fetal head using a light transducer pressure and color Doppler. The insonation angle was kept as close to zero degrees as was achievable. The MCA PI was recorded just distal to the circle of Willis, the ACA PI was recorded in the first segment distal to the junction with the internal carotid artery, and the PCA PI was recorded in the second segment distal to the junction with the posterior communicating artery. The VertA was identified in the nuchal region using the color Doppler and was recorded at its location between the first cervical vertebra and the occipital bone. The UA PI was measured from a free loop of cord with the insonation angle < 30°. The UV diameter was recorded in a transverse section of a magnified image with calipers placed at the inner edges of the vessel wall. The UtA PI was recorded at the level of the maternal iliac vessels, and the mean of the left and right uterine arteries was used.

The cerebroplacental ratio (CPR) was calculated as the ratio of the MCA PI to the UA PI. Ratios were also generated for the other cerebral vessels to the UA PI, namely the ACA PI/UA PI, PCA PI/UA PI, and VertA PI/UA PI. UV flow in milliliters per minute was calculated using the following formula: Time Averaged Velocity (centimeters per second) × 0.3 × cross-sectional area of the vessel (square millimeters) corrected for estimated fetal weight in kilograms [22].

Fetal CO (Left (LCO), Right (RCO), and Combined (CCO)) was calculated using a formula [23] incorporating the stroke volume (SV); time-velocity integral (TVI), obtained by manually tracing the pulse wave waveform, from the left and right outflow tracts, respectively [24]; pulmonary or aortic valve radius (r), respectively; and fetal heart rate (FHR) and corrected for EFW (mL/min/kg):

SV (ml) = π × r^2^ × TVI = π × (valve diameter/2)^2^ × TVI (1)

CO (mL/min) = SV × FHR(2)

All obstetric caregivers and participating women were blinded to the ultrasound findings unless a malpresentation or small for gestational age (EFW < 5th centile) or large for gestational age (EFW > 95th centile) fetus was detected. Maternal demographic data and intrapartum and neonatal outcomes were extracted from the electronic hospital database. Indications for operative birth were recorded as those made contemporaneously by the treating obstetric team. A diagnosis of intrapartum fetal compromise (IFC) was made either on the basis of a pathological FHR pattern or an abnormal fetal scalp lactate (>4.2 mmol/L). Neonatal acidosis was defined as a cord artery pH < 7 or base excess of <−12mmol/L. EFW centiles were based on an Australian population and corrected for gender and gestational age [25]. The CPR centiles were also based on an Australian population and corrected for gestational age [26].

### Statistical Analysis

Sample size calculation was performed by conservatively estimating that the mean MCA PI for gestation would decrease by approximately 10% (from 1.71 to 1.54) at a gestation of 36 weeks in women who slept in a supine position [26]. Using a standard deviation of 0.25, an alpha of 0.05, and a power of 0.9, a sample size of 46 in each group was required.

The statistical software package Stata, Release 13, for Windows (StataCorp LP, College Station, TX, USA) was used to perform the statistical analysis.

Demographic characteristics were summarized using mean and standard deviation for normally distributed continuous variable, median and interquartile range for non-normally distributed continuous variables, and frequency and percent for categorical variables. A logistic regression model was used to examine the association between supine sleep and ultrasound parameters, as well as perinatal outcomes after adjusting for relevant demographic factors.

## 3. Results

**Figure 1:**
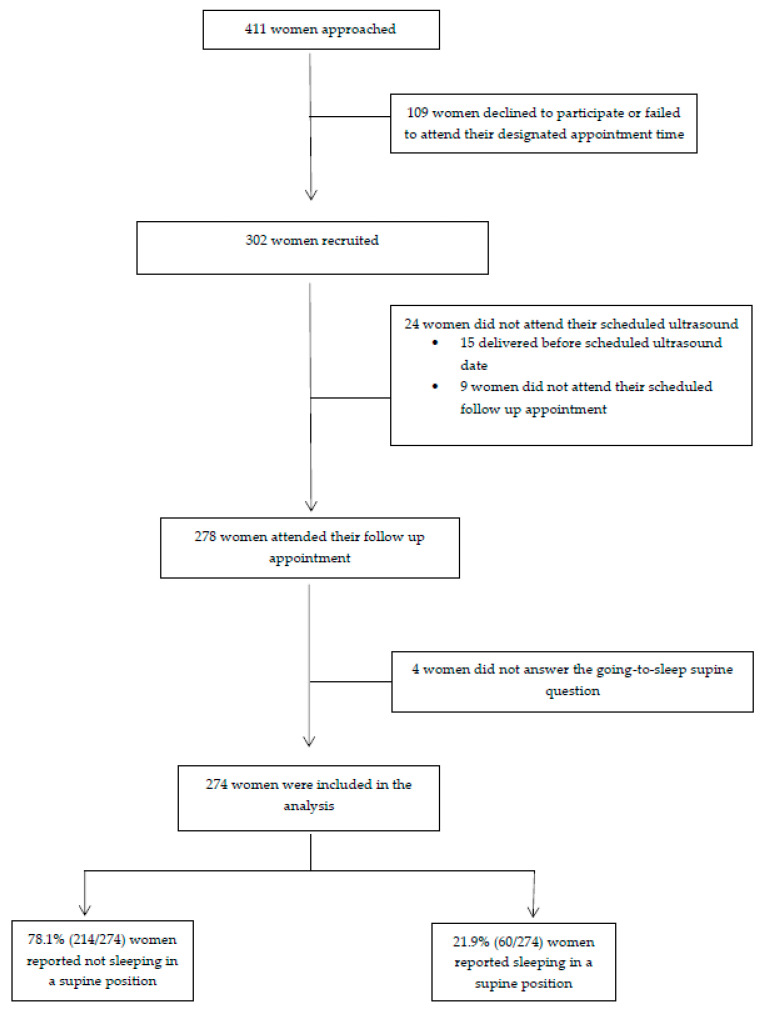
Recruitment flowchart.

A total of 411 women met the inclusion criteria and were approached to participate in this study. Although 302 women were eventually recruited, only 274 women answered the complete questionnaire and underwent the ultrasound scan and were included in the final analysis (Figure 1). Overall, 78.1% (214/274) of participants were in the control group and 21.9% (60/274) were in the supine cohort. There were no significant demographic differences between the two groups (Table 1).

**Figure 2:**
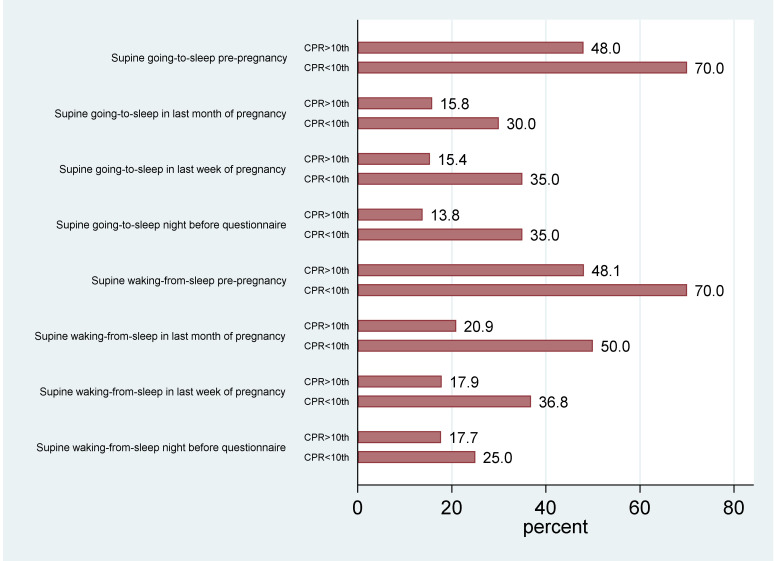
Maternal supine sleep position and cerebroplacental ratio (CPR) centile.

Table 2 shows the differences for the various ultrasound parameters between the two groups. There were three cerebral parameters that were significantly lower in the supine cohort: The mean MCA PI (1.62 (0.25) vs. 1.74 (0.29), *p* = 0.001), ACA PI (1.52 (0.27) vs. 1.59 (0.30). *p* = 0.046), and VertA PI (1.57 (0.27) vs. 1.68 (0.35), *p* = 0.019). The CPR was also lower in the supine sleep cohort (1.98 (0.37) vs. 2.13 (0.42); *p* = 0.008) (Figure 2 and Supplementary Table S2). There were however no differences in the UA PI or the ACA/UA, PCA/UA, or VertA/UA ratios. There were also no differences in UtA PI, UV flow, EFW, or EFW centile.

Additionally, there were no differences in the fetal cardiac output parameters between the cohorts. The LCO, RCO, and CCO were all lower in the supine sleep cohort (even when corrected for fetal weight) but did not reach statistical significance: Mean LCO (465 mL/min (118.6) vs. 492.5 mL/min (147.0), *p* = 0.30), RCO (758.93 mL/min (178.37) vs. 778.67 mL/min (189.22), *p* = 0.68), CCO (1223.9 mL/min (266.5) vs. 1271.1 mL/min (297.9), *p* = 0.44).

Table 3 details the obstetric and neonatal outcomes between the two cohorts. There were no differences in mode of birth, with similar rates of spontaneous vaginal delivery (56.3% (120/213) vs. 66.7% (40/60), *p* = 0.20]) elective caesarean section (8.9% (19/213) vs. 5.0% (3/60), *p* = 0.35), instrumental delivery (18.3% (39/213) vs. 15.0% (9/60), *p* = 0.64), and emergency caesarean section (16.4% (35/213) vs. 13.3% (8/60), *p* = 0.59) between the control and supine sleep groups, respectively. There was also no difference observed in the indication for operative delivery or serious neonatal outcomes between the two groups. Women in the supine cohort were more likely to have an infant with a BW > 90th centile (*p* = 0.04).

## 4. Discussion

The results of this study demonstrate significant differences in fetal cerebral Doppler indices in nonobese women who slept in a supine position in the third trimester of pregnancy. Specifically, we found that the MCA PI, CPR, ACA PI, and VertA PI were all lower in the supine sleep position cohort consistent with cerebral redistribution.

Although the fetal MCA, due to the ease of its imaging, is the cerebral vessel most commonly reported in Doppler studies of fetal wellbeing, there is evidence that cerebral redistribution occurs in a regional, stepwise temporal fashion with early changes occurring in the frontal lobe. The PCA supplies the occipital brain, and changes in this vessel tend to occur later in the redistributive process [27]. Changes in the ACA [27,28] and VertA/UA ratio [29,30] have been shown to be associated with adverse perinatal outcomes or suboptimal fetal growth, although there is a lack of evidence demonstrating its superiority over other parameters of fetal wellbeing [30,31]. The differences in cerebral perfusion are interesting given the lack of fetal or birth weight discordance between our cohorts, suggesting that the maternal supine sleep position may cause subtle circulatory changes independent of perturbations in growth that may increase the vulnerability of these fetuses to adverse outcomes. The lack of difference in UA PI suggests no overt degree of placental dysfunction, however the altered cerebral vascular indices suggest that there is some degree of compensation occurring within the fetus. The lack of evidence of systemic alterations in perfusion could also be due to the low-risk nature of the cohort, which was reflected in the fact there were no differences in perinatal outcomes between cohorts.

Interestingly, women in the supine sleep cohort were more likely to have an infant with a BW > 90th centile. Although the literature regarding altered fetal growth and maternal sleep is conflicting, increased birth weight has been previously reported in women with SDB [32,33,34,35]. Nevertheless, given the relatively limited numbers of women in this study, it is possible that this particular finding may have arisen by chance.

As the fetal brain sparing effect is mediated by alterations in cardiac output [24,36], we sought to ascertain if we could detect any changes in cardiac output and correlate this with regional cerebral perfusion. Fetal cardiac function has been shown to be impaired in growth restricted cohorts [37,38], as well as appropriately grown fetuses that go on to develop IFC [39]. We previously showed that term fetuses that develop IFC have lower LCO and higher RCO. More recent data have suggested that the CPR, MCA PI, and UV flow are also associated with alterations in fetal cardiac parameters, including the LCO [40]. However, the same study demonstrated that only around 17% of the CPR could be explained by alterations in cardiac indices [40]. The mechanisms underpinning cardiac adaptations to a hostile intrauterine environment are complex. Given that the fetuses in our study were not small, it is possible that the cerebral blood flow changes we identified were mediated more by vascular homeostatic mechanisms (changes in resistance, etc.), rather than more overt cardiac mechanisms. Given that overall our cohort was normally grown and low risk, it is possible that the alterations were too subtle to detect, or that our sample size was not large enough given that it was powered based on MCA Doppler changes.

The rate of supine sleep in our cohort was 21.9%, which is consistent with rates reported by other investigators [1,2]. Warland et al. proposed that altered uterine perfusion as a result of supine positioning may be relevant in a scenario where the fetus is already ‘vulnerable’ to hypoxic stress as a result of other factors being present. These may include maternal demographic risk factors, co-morbid conditions, or the presence of pre-existing impaired placental function [41]. Other work has demonstrated that maternal position affects fetal behavioral states and heart rate variability [42] and is a modifiable risk factor that could be targeted to decrease the incidence of stillbirth. Indeed, a recent publication indicated that intervention aimed at reducing time spent in a supine sleep position was feasible, improved maternal and fetal parameters, and did not negatively impact maternal sleep quality [43]. Another recent publication suggested that the supine position was not associated with stillbirth [44], but there were various methodological differences that could have accounted for this alternate finding [45], the most significant of which was the gestation at assessment being earlier when the gravid uterus had less of an impact on maternal circulation.

One of the constraints of this study was the use of the self-reported sleep position [46]. However, this is an accepted method of determining sleep position in pregnancy and has been used in other large studies notwithstanding its limitations [3,4,5,7,47]. The going-to-sleep position using self-reported questionnaires has good concordance with video surveillance of the maternal sleep position [48]. Our rationale for using the going-to-sleep position was also relevant, as it is a potentially modifiable risk factor as opposed to the maternal waking-from-sleep position. Reporting women who sleep supine less than 10% of the night with women who sleep supine ≥ 10% of the night may also be a limitation of this study if the fetal response is considered to be dose-dependent. However, in our view, the physiological changes associated with pregnancy in the third trimester would be more magnified when the gravid uterus is at its heaviest, so any time spent in a supine position would have an effect, hence our rationale for including all women who reported sleeping supine ≥ 10% of the time. The use of data from a single time point is also a possible limitation, however the difficulty of pinpointing the exact gestation at which supine sleep began makes a pre-supine sleep assessment difficult to obtain, particularly if a woman sleeps in a supine position throughout pregnancy. The use of a robust sample size, prospective study design, and comprehensive fetal Doppler and cardiac assessment were strengths of this study. As our study was exploratory and hypotheses-generating, it was not powered to demonstrate differences in obstetric or perinatal outcomes. Interestingly, Dunietz et al. [49] objectively assessed women in the third trimester for sleep position, maternal respiratory events, and perinatal outcomes. This study also found no association between maternal supine sleep position and perinatal outcomes. However, it did find an association between maternal supine sleep and more maternal respiratory and oxygen desaturation events, which may account for the fetal redistribution pattern observed in this study.

The data presented in this manuscript provides information regarding some of the fetal circulatory changes in women who sleep in the supine position during late pregnancy and may provide some insight into the mechanisms associated with late stillbirth and adverse obstetric and perinatal outcomes in these women given the association between a low CPR and poor outcomes [50,51,52].

Our results are pertinent because it is now well known that the majority of late stillbirths, particularly at term, are not small or growth-restricted [53]. Indeed, there is now considerable evidence that fetal cerebral redistribution is associated with adverse outcomes, including perinatal death, even in appropriately grown fetuses [29,54,55,56]. In animal models, it has been proposed that the regulation of cerebral blood flow is area-specific, with some areas more prone to cerebral injury in the event of a hypoxic event [57,58]. It has also been proposed that fetal brain injury may actually be initiated as a consequence of cerebral redistribution, with increased flow as a particular risk factor [59,60]. Animal models exhibiting the same brain sparing effect as human fetuses [61] have demonstrated that pregnant mice experiencing an acute hypoxic event are particularly vulnerable to any further hypoxic injury leading to fetal death [62]. Extrapolating animal and other human data to the context of supine sleep position suggest that similar mechanisms could also be responsible for the increased risk of stillbirth seen in women who sleep on their backs. Further studies are clearly required to elucidate fetal and maternal physiological changes potentially linking supine sleep position to fetal compromise and death.

Our findings and those of others [20,63] suggest that some degree of cerebral redistribution occurs in the maternal supine sleep position. Furthermore, there is strong epidemiological evidence that the supine sleep position may be causative for some adverse outcomes, including stillbirth. Given that cerebral redistribution is a physiological response to fetal compromise [64], it is tempting to postulate that supine sleep causes placental dysfunction from the reduction in placental perfusion and results in the subsequent change in fetal hemodynamics. However, in our view, the evidence for such extrapolation is limited and the association should not be considered causative at this stage. Public health advice in many jurisdictions already cautions women against sleeping on their back during pregnancy. Our findings provide further evidence of some of the potential physiological mechanisms underpinning the increased perinatal risks associated with the supine sleep position. However, we are unable to provide any advice regarding the frequency of ultrasound surveillance or timing of birth for women who spend the majority of time sleeping in the supine position, and further research is required.

## Figures and Tables

**Table 1:** Maternal demographics by maternal sleep position.

Variable	No Supine Sleep *n* = 214 (78.1%)	Supine Sleep *n* = 60 (21.9%)	*p* Value
Maternal age (mean, SD) †	31.5 (4.2)	31.0 (3.8)	0.43
Gestational age at ultrasound assessment (weeks) (mean, SD) †	36.4 (0.8)	36.2(0.6)	0.06
Maternal booking BMI (median, IQR) ‡	23.3 (21.1–27.1)	23.5 (20.9–28.3)	0.99
Ethnicity §			0.11
Caucasian	64.5% (138/214)	53.3% (32/60)	
ATSI	0.9% (2/214)	1.7% (1/60)	
Asian	15.9% (34/214)	13.3% (8/60)	
Indian	8.4% (18/214)	20.0% (12/60)	
Other	10.3% (22/214)	11.7% (7/60)	
Parity §			0.93
Nulliparous	40.6% (87/214)	40.0% (24/60)	
Multiparous	59.4% (127/214)	60.0% (36/60)	
Smoking §	27.6% (59/214)	25.0% (15/60)	0.69
Hypertension ∆	3.8% (8/213)	5.1% (3/59)	0.71
Diabetes §	15.0% (32/213)	13.6% (8/59)	0.78

†: Student *t*-test; ‡: Mann-Whitney U-test; §: Chi-squared test; **∆**: Fisher’s exact test. BMI: Body mass index; ATSI: Aboriginal and Torres Strait Islander; SD: Standard deviation; IQR: Interquartile range.

**Table 2:** Fetal Doppler and cardiac parameters by maternal sleep position.

Parameter	No Supine Sleep *n* = 214 (78.1%)	Supine Sleep *n* = 60 (21.9%)	*p* Value *
MCA PI	1.74 (0.29)	1.62 (0.25)	0.001
UA PI	0.83 (0.12)	0.83 (0.12)	0.81
CPR	2.13 (0.42)	1.98 (0.37)	0.008
CPR < 10th centile	6.1% (13/214)	11.7% (7/60)	0.16
ACA PI	1.59 (0.30)	1.52 (0.27)	0.046
PCA PI	1.48 (0.28)	1.42 (0.23)	0.055
VertA PI	1.68 (0.35)	1.57 (0.27)	0.019
ACA/UA ratio	1.94 (0.43)	1.87 (0.41)	0.19
PCA/UA ratio	1.80 (0.38)	1.74 (0.34)	0.15
VertA/UA ratio	2.04 (0.48)	1.89 (0.42)	0.06
LCO	492.50 (147.02)	465.00 (118.61)	0.30
^#^ LCO (mL/min/kg)	167.03 (44.38)	161.82 (38.68)	0.39
RCO	778.67 (189.22)	758.93 (178.37)	0.68
^#^ RCO (mL/min/kg)	264.50 (57.56)	264.58 (60.52)	0.92
LCO/RCO ratio	0.64 (0.16)	0.62 (0.13)	0.38
CCO	1271.15 (297.86)	1223.93 (266.50)	0.44
^#^ CCO (mL/min/kg)	431.59 (87.29)	426.39 (88.66)	0.62
UtA PI	0.68 (0.17)	0.71 (0.22)	0.23
UV flow (mL/min/kg)	84.61 (25.94)	81.89 (27.96)	0.31
EFW (grams)	2935.46 (332.75)	2877.58 (287.51)	0.79
EFW centile	52.73 (23.91)	50.83 (24.49)	0.65

* Adjusted for gestational age at ultrasound assessment; ^#^ Adjusted for EFW; MCA PI: Middle cerebral artery pulsatility index; UA PI: Umbilical artery pulsatility index; CPR: Cerebroplacental ratio; ACA PI: Anterior cerebral artery pulsatility index; PCA PI: Posterior cerebral artery pulsatility index; VertA PI: Vertebral artery pulsatility index; LCO: Left cardiac output; RCO: Right cardiac output; CCO: Combined cardiac output; UtA PI: Uterine artery pulsatility index; UV: Umbilical vein; mL/min/kg: Milliliters per minute per kilogram; EFW: Estimated fetal weight.

**Table 3:** Intrapartum and perinatal outcomes by maternal sleep position.

Outcome	No Supine Sleep *n* = 214 (78.1%)	Supine Sleep *n* = 60 (21.9%)	*p* Value *
Gestation at delivery (weeks) (median, IQR)	39.3(38.6–40.3)	39.6 (38.9–40.4)	0.21
Mode of birth			
SVD	56.3% (120/213)	66.7% (40/60)	0.20
Elective CS	8.9% (19/213)	5.0% (3/60)	0.35
Instrumental all	18.3% (39/213)	15.0% (9/60)	0.64
Em CS all	16.4% (35/213)	13.3% (8/60)	0.59
Em CS IFC	7.5% (16/213)	5.0% (3/60)	0.51
Em CS Other	8.9% (19/213)	8.3% (5/60)	0.92
Perinatal outcomes			
BW grams (median, IQR)	3384 (3084–3680)	3419 (3124–3745)	0.66
BW <10th centile	12.2% (26/213)	13.3% (8/60)	0.93
BW >90th centile	5.2% (11/213)	13.3% (8/60)	0.04
5-min Apgar < 7	1.4% (3/211)	3.4% (2/59)	0.24
Acidosis (pH < 7 or BE <−12)	1.9% (4/213)	1.7% (1/60)	0.95
NICU admission	5.2% (11/211)	5.0% (3/60)	0.95

* Adjusted for gestational age at ultrasound assessment; IQR: Interquartile range; SVD: Spontaneous vaginal delivery; CS: Cesarean section; IFC: Intrapartum fetal compromise; Em CS: Emergency cesarean section; BW: Birth weight; BE: Base excess; NICU: Neonatal intensive care unit.

## Supplementary Materials

The following are available online at https://www.mdpi.com/2077-0383/9/6/1773/s1, Table S1. Sleep questionnaire, Supplementary Table S2: Maternal demographics by cerebral redistribution
