# Supplementary material for: Sleep in the Supine Position During Pregnancy is Associated with Fetal Cerebral Redistribution
Source: J Clin Med. 2020 Jun 7;9(6):1773. doi: 10.3390/jcm9061773 (PMC7356729; doi:10.3390/jcm9061773)
Supplement: Supplementary file 1 [file jcm-09-01773-s001.pdf]

Supplementary Materials: Sleep in the Supine Position During Pregnancy is Associated with Fetal Cerebral Redistribution

Table S1. Sleep questionnaire.

Question		Possible responses
Position on going to sleep before you were pregnant?	Front	No / Yes / Rarely (<10%) / Sometimes (10–50%) / Mostly (>50%)
	Back	No / Yes / Rarely (<10%) / Sometimes (10–50%) / Mostly (>50%)
	Left side	No / Yes / Rarely (<10%) / Sometimes (10–50%) / Mostly (>50%)
	Right side	No / Yes / Rarely (<10%) / Sometimes (10–50%) / Mostly (>50%)
	Unsure	No / Yes
Position on going to sleep in the past one month of pregnancy?	Front	No / Yes / Rarely (<10%) / Sometimes (10–50%) / Mostly (>50%)
	Back	No / Yes / Rarely (<10%) / Sometimes (10–50%) / Mostly (>50%)
	Left side	No / Yes / Rarely (<10%) / Sometimes (10–50%) / Mostly (>50%)
	Right side	No / Yes / Rarely (<10%) / Sometimes (10–50%) / Mostly (>50%)
	Unsure	No / Yes
Position on going to sleep in the past one week of pregnancy?	Front	No / Yes / Rarely (<10%) / Sometimes (10–50%) / Mostly (>50%)
	Back	No / Yes / Rarely (<10%) / Sometimes (10–50%) / Mostly (>50%)
	Left side	No / Yes / Rarely (<10%) / Sometimes (10–50%) / Mostly (>50%)
	Right side	No / Yes / Rarely (<10%) / Sometimes (10–50%) / Mostly (>50%)
	Unsure	No / Yes
Position on going to sleep during last night?	Front	No / Yes / Rarely (<10%) / Sometimes (10–50%) / Mostly (>50%)
	Back	No / Yes / Rarely (<10%) / Sometimes (10–50%) / Mostly (>50%)
	Left side	No / Yes / Rarely (<10%) / Sometimes (10–50%) / Mostly (>50%)
	Right side	No / Yes / Rarely (<10%) / Sometimes (10–50%) / Mostly (>50%)
	Unsure	No / Yes
Position on waking up before you were pregnant?	Front	No / Yes / Rarely (<10%) / Sometimes (10–50%) / Mostly (>50%)
	Back	No / Yes / Rarely (<10%) / Sometimes (10–50%) / Mostly (>50%)
	Left side	No / Yes / Rarely (<10%) / Sometimes (10–50%) / Mostly (>50%)
	Right side	No / Yes / Rarely (<10%) / Sometimes (10–50%) / Mostly (>50%)
	Unsure	No / Yes
Position on waking up in the past one month of pregnancy	Front	No / Yes / Rarely (<10%) / Sometimes (10–50%) / Mostly (>50%)
	Back	No / Yes / Rarely (<10%) / Sometimes (10–50%) / Mostly (>50%)
	Left side	No / Yes / Rarely (<10%) / Sometimes (10–50%) / Mostly (>50%)
	Right side	No / Yes / Rarely (<10%) / Sometimes (10–50%) / Mostly (>50%)
	Unsure	No / Yes
Position on waking up in the past one week of pregnancy?	Front	No / Yes / Rarely (<10%) / Sometimes (10–50%) / Mostly (>50%)
	Back	No / Yes / Rarely (<10%) / Sometimes (10–50%) / Mostly (>50%)
	Left side	No / Yes / Rarely (<10%) / Sometimes (10–50%) / Mostly (>50%)
	Right side	No / Yes / Rarely (<10%) / Sometimes (10–50%) / Mostly (>50%)
	Unsure	No / Yes
Position on waking up during last night	Front	No / Yes / Rarely (<10%) / Sometimes (10–50%) / Mostly (>50%)
	Back	No / Yes / Rarely (<10%) / Sometimes (10–50%) / Mostly (>50%)
	Left side	No / Yes / Rarely (<10%) / Sometimes (10–50%) / Mostly (>50%)
	Right side	No / Yes / Rarely (<10%) / Sometimes (10–50%) / Mostly (>50%)
	Unsure	No / Yes

Table S2. Maternal demographics by cerebral redistribution.

Variable	CPR < 10th centile *n=20 (7.3%)*	CPR > 10th centile *n=254 (92.7%)*	p value
Maternal age (mean, SD) †	31.4 (2.1)	31.4 (4.3)	0.97
Gestational age at ultrasound assessment (weeks) (mean, SD) †	36.3 (1.0)	36.4 (0.7)	0.55
Maternal booking BMI (median, IQR) ‡	24.99 (22.02–28.81)	23.18 (21.09–27.13)	0.03
Ethnicity¶			0.74
Caucasian	55% (11/20)	62.6% (159/254)	
ATSI	0	1.2% (3/254)	
Asian	15% (3/20)	15.4% (39/254)	
Indian	15% (3/20)	10.6% (27/254)	
Other	15% (3/20)	10.2% (26/254)	
Parity§			0.052
Nulliparous	20.0% (4/20)	42.1% (107/254)	
Multiparous	80.0% (16/20)	57.9% (147/254)	
Smoking§	20.0% (4/20)	27.6% (70/254)	0.46
Hypertension¶	10.0% (2/20)	3.6% (9/252)	0.19
Diabetes¶	25.0% (5/20)	13.9% (35/252)	0.19

† Student t-test; ‡ Mann-Whitney U-test; § Chi-squared test; ¶Fisher's exact test. CPR: cerebroplacental ratio; BMI: body mass index; ATSI: Aboriginal and Torres Strait Islander; SD: standard deviation; IQR: interquartile range.

© 2020 by the authors. Licensee MDPI, Basel, Switzerland. This article is an open access article distributed under the terms and conditions of the Creative Commons Attribution (CC BY) license (http://creativecommons.org/licenses/by/4.0/).
